# miRNAs involved in LY6K and estrogen receptor α contribute to tamoxifen-susceptibility in breast cancer

**DOI:** 10.18632/oncotarget.9950

**Published:** 2016-06-11

**Authors:** Ye Sol Kim, Sae Jeong Park, Yeon Seon Lee, Hyun Kyung Kong, Jong Hoon Park

**Affiliations:** ^1^ Department of Biological Science, Sookmyung Women's University, Seoul, Korea

**Keywords:** breast cancer, tamoxifen susceptibility, LY6K, ERα, miRNA

## Abstract

Estrogen receptor-alpha (ERα) is a clinically important therapeutic target for breast cancer. However, tumors that lose ERα are less responsive to anti-estrogens such as tamoxifen. MicroRNAs (miRNAs) are small RNAs that regulate expression of their target gene and dysregulations of miRNA has been identified in many diseases including human cancer. However, only a few miRNAs associated with tamoxifen resistance has been reported. In this study, we found that lymphocyte antigen 6 complex (LY6K), which is a member of the Ly-6/μPAR superfamily and related to breast cancer progression and metastasis, is inversely correlated with ERα expression. We, for the first time, found miRNAs involved in the regulatory molecular mechanism between ERα and LY6K and related to tamoxifen susceptibility in breast cancer. miR-192-5p, induced by LY6K, downregulates ERα directly and induced tamoxifen resistance in ERα-positive breast cancer cells. In addition, re-expression of ERα in ERα-negative breast cancer cells increased miR-500a-3p expression and directly inhibits LY6K expression. Ectopic expression of miR-500a-3p sensitized ERα-negative cells to tamoxifen by increasing apoptosis. Finally, we observed an inverse correlation between LY6K and ERα in primary breast cancer samples. We found that patients with recurrence showed high expression of miR-192-5p after tamoxifen treatments. In addition, expression of miR-500a-3p was significantly correlated to survival outcome. As miRNAs involved in the regulatory mechanism between LY6K and ERα can affect tamoxifen resistance, downregulating miR-192-5p or re-expressing miR-500a-3p could be a potential therapeutic approach for treating tamoxifen resistant patients.

## INTRODUCTION

Breast cancer is the common malignant tumor world-wide in women. Classically, breast cancer is classified into several subgroups according to the expression of the following receptors: estrogen receptor (ER), progesterone receptor (PR) and human epidermal growth factor receptor 2 (HER2) [1]. Approximately 70% of breast cancer patients are dependent on estrogen receptor α (ERα) [2], so diverse treatment options have been developed targeting ERα [3], [4]. Tamoxifen is broadly used in adjuvant treatment for ER-positive breast cancer patients. In addition, Tamoxifen was clinically associated with growth arrest and apoptosis by interrupting estrogen binding to the ER in ERα-positive breast cancer. However, tumors that undergo loss of ER or negative expression of ER have less responsiveness to ER-targeted drugs in breast cancer [5].

Lymphocyte antigen 6 complex locus K (LY6K) is a member of the Ly-6/urokinase-type plasminogen activator receptor (uPAR) superfamily [6]. Recently, LY6K was reported to be a molecular biomarker in breast cancer [7], bladder cancer [8] and esophageal squamous cell carcinomas [9]. In addition, LY6K expression is regulated by the AP-1 transcription factor that promotes cell proliferation, invasive and metastatic abilities in breast cancer cells [10]. Nonetheless, there are few studies to supporting the effect of LY6K on various type of breast cancer.

A variety of cellular activities are regulated by MicroRNAs (miRNAs) such as apoptosis, metabolism and proliferation in many cancer types [11, 12]. Recent studies have demonstrated that the expression of miRNAs is related to pathologic features in breast cancer [13]. miR-221/222 [14, 15] and miR-181 [16] contribute to the development of tamoxifen resistance by degrading genes involved in the regulation of breast cancer therapies. Furthermore, re-expression of miR-320a [17], miR-375 [18], and miR-342 [19] could restore tamoxifen sensitivity by inhibiting their targets.

In this study, we found an inverse correlation between the expression of ERα and LY6K in breast cancer. By investigating the molecular mechanism behind the functions of miR-192-5p and miR-500a-3p in the regulation of ERα and LY6K, how miR-192-5p, induced by LY6K, causes tamoxifen resistance by inhibiting ERα expression in ERα-positive breast cancer. In addition, we elucidated the mechanism miR-500a-3p is induced by ERα and downregulates LY6K expression and re-expression of miR-500a-3p sensitizes tamoxifen responsivity. Our findings provides a rationale for downregulating miR-192-5p or re-expressing miR-500a-3p as a potential therapeutic strategy for treating tamoxifen resistant patients.

## RESULTS

### LY6K and ERα is negatively expressed in breast cancer cells

**Figure 1:**
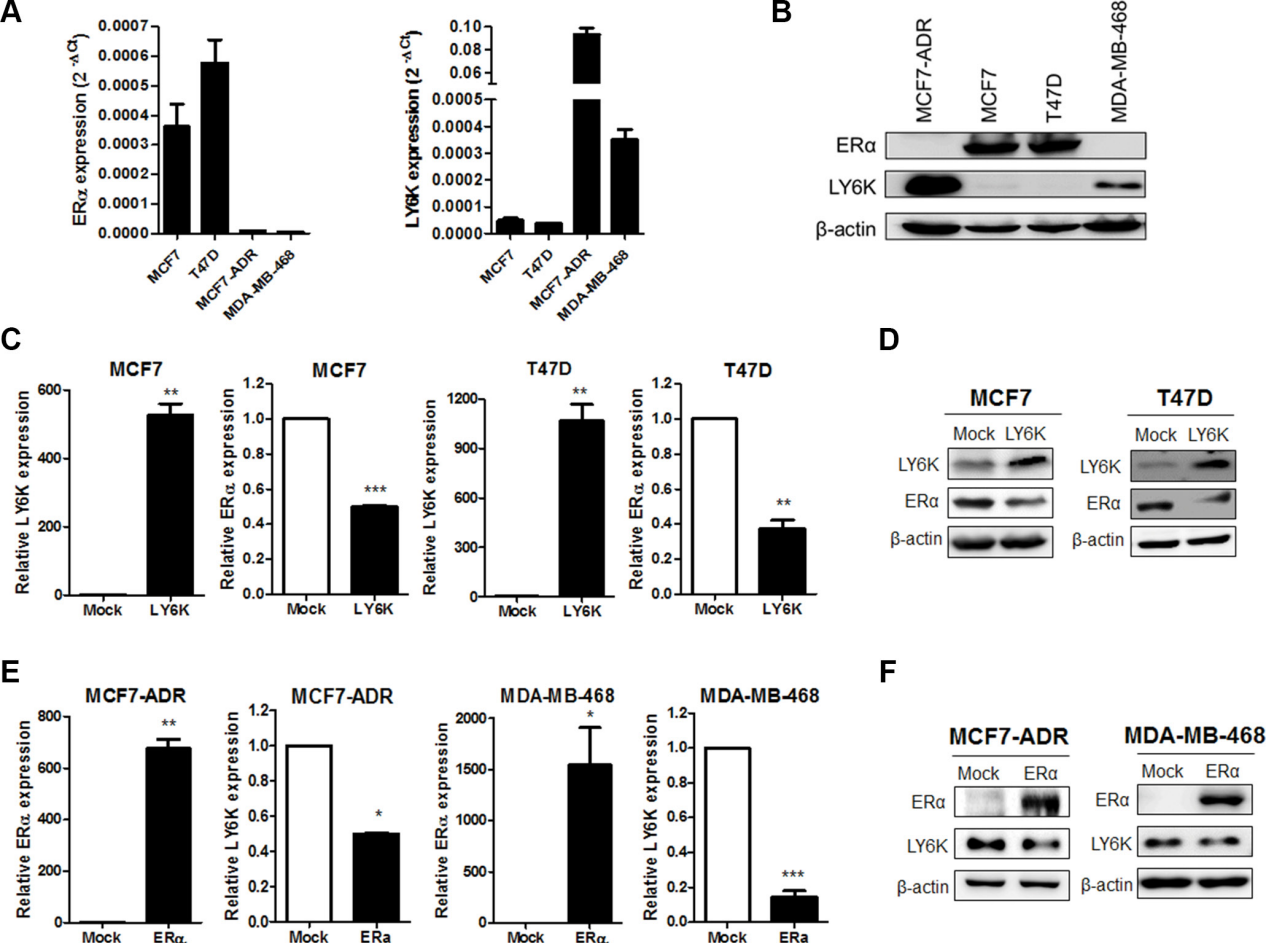
The expression of ERα is negatively correlated with LY6K (**A** and **B**) Expression of LY6K and ERα mRNA and protein was observed by qRT-PCR using specific primers and western blotting. h18s rRNA and β-actin were used for endogenous control. (**C** and **D**) Ectopic expression of LY6K in MCF7 and T47D downregulated ERα transcriptional activity and protein expression. (**E** and **F**) Ectopic expression of ERα in MCF7-ADR and MDA-MB-468 reduce LY6K mRNA and protein expression. Data are a mean ± S.D. (error bars) of three independent experiment in triplicate. ***p < 0.001; **p < 0.01; *p < 0.05.

To investigate LY6K is involved in the regulation of ERα expression, we evaluated both the mRNA and protein levels in the breast cancer cell lines. We confirmed that the ERα-positive breast cancer cell lines, MCF7 and T47D, did not express LY6K mRNA and protein, whereas the ERα-negative breast cancer cell lines, MCF7-ADR and MDA-MB-468, expressed LY6K mRNA and protein (Figure 1A and 1B). To confirm the inverse correlation between ERα and LY6K, we observed ERα expression by overexpressing LY6K in ERα-positive breast cancer cell lines. Both ERα mRNA and protein were downregulated by LY6K (Figure 1C and 1D). In addition, we observed that the expression of LY6K was reduced by ectopic expression of ERα in MCF7-ADR and MDA-MB-468, well known as ERα-negative breast cancer cells. Ectopic expression of ERα led to the reduction of both LY6K mRNA and protein expression in ERα-negative breast cancer cells (Figure 1E and 1F).

### LY6K and ERα expression is regulated in miRNA-dependent manner

**Figure 2:**
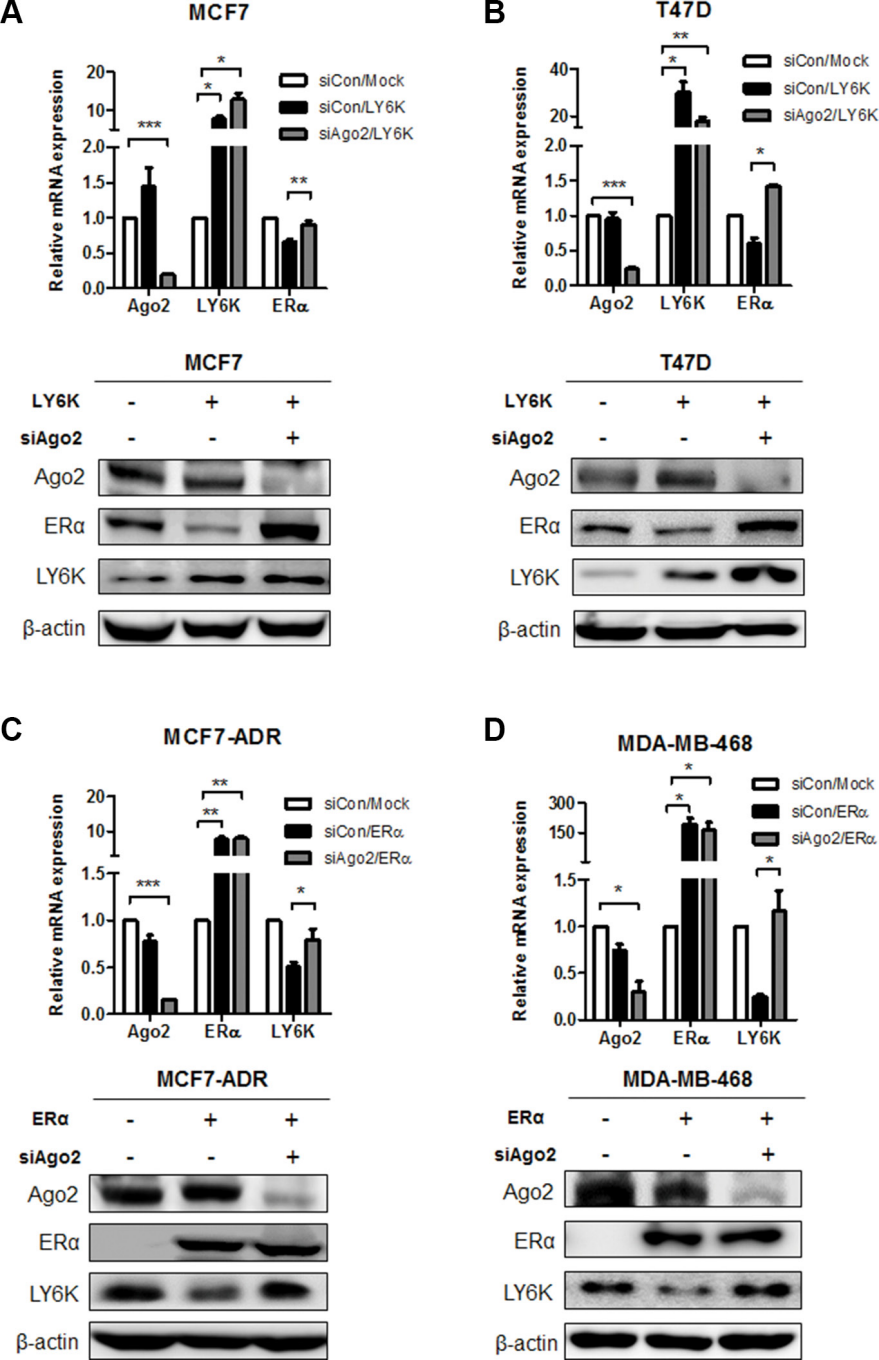
The regulation of ERα and LY6K expression in a miRNA-dependent manner (**A** and **B**) Gene expression level was determined on both mRNA and Protein levels after treatment with Ago2 siRNA combined with overexpression of LY6K for 48 hr. The expression of mRNA and protein was analyzed by qRT-PCR and western blotting. (**C** and **D**) LY6K expression in response to knock-down of AGO2 by treating with control and ERα for 48 hr. The expression of mRNA and protein was investigated by qRT-PCR and western blotting. Data are a mean ± S.D. (error bars) of three independent experiment in triplicate. ***p < 0.001; **p < 0.01; *p < 0.05.

To identify the molecular events involved in the inverse correlation between LY6K and ERα, we hypothesized that the upregulation of miRNA by LY6K and ERα might mediate the expression of target genes. To verify this hypothesis, we confirmed the level of ERα expression level through simultaneously the overexpression of LY6K and knock-down of Argonate 2 (Ago2), which participates in mature miRNA processing as a member of the RNA-induced silencing (RISC) complex protein. As a result, the mRNA and protein expression level for ERα reduced by ectopic expression of LY6K, whereas knock-down of Ago2 restored the expression of ERα (Figure 2A and 2B). Furthermore, ectopic expression of ERα led to the reduction of LY6K expression but AGO2 knockdown did not further reduce LY6K expression in MCF7-ADR, MDA-MB-468 (Figure 2C and 2D). Collectively, these results indicated that the regulation mechanism for LY6K and ERα is dependent on miRNAs.

### LY6K and ERα affect miRNA expression in breast cancer cells

On the basis of these observations, we further investigate to identify miRNAs affected by the overexpression of LY6K or ERα. We first carried out miRNA microarray analysis in T47D, ERα-positive cells, compared with T47D/LY6K (Supplementary Figure S1A). Another miRNA microarray analysis was performed in comparison with MCF7-ADR, ERα-negative cells, MCF7-ADR/ERα, transiently overexpress ERα. (Supplementary Figure S1B). As a result, we found several miRNA which were affected and upregulated by LY6K or ERα. To search for miRNAs which directly target to ERα and LY6K 3′ untranslated region (3′UTR), we used miRanda (http://www.microrna.org) computational tools and identified candidate miRNAs.

**Figure 3:**
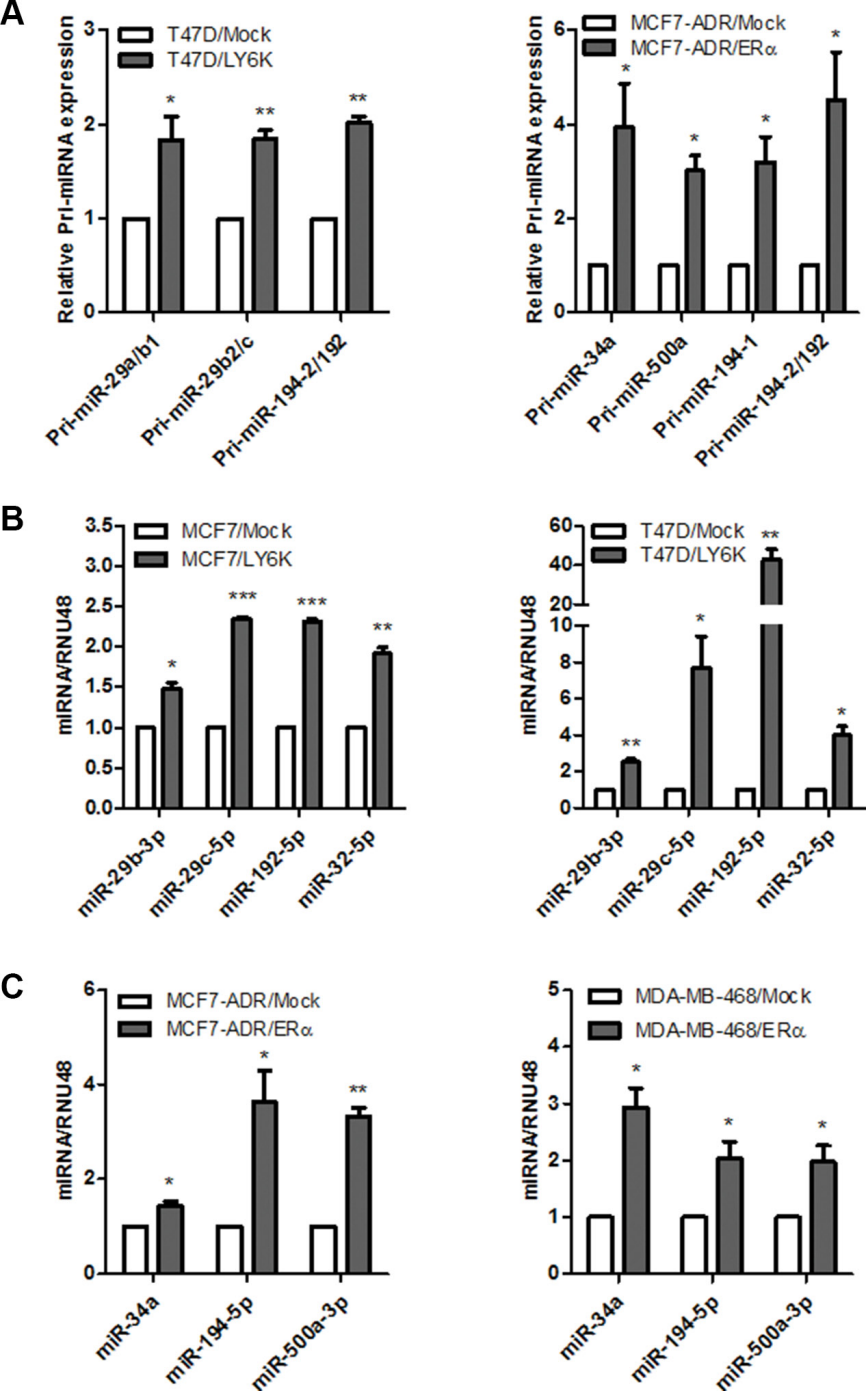
Validation of primary and mature miRNAs induced by LY6K or ERα (**A**) Validation of upregulated primary miRNA (pri-miRNA) expression in T47D/LY6K was compared with T47D/Mock (left) and MCF7-ADR/Mock compared with MCF7-ADR/ERα (right) by using qRT-PCR. Each primary miRNA produces mature miRNAs (Pri-miR-29a/b1: miR-29b, Pri-miR29b2/c: miR-29b and miR-29c, Pri-miR-194-2/192: miR-192-5p and miR-194, Pri-miR-194-1: miR-194, Pri-miR-34a: miR-34a, Pri-miR-500a: miR-500a-5p or -3p). (**B**) Verification of miRNA expression induced by LY6K in ERα-positive breast cancer cell lines, MCF7 (left) and T47D (right). (**C**) Verification of mature miRNA induced by ERα in ERα-negative breast cancer cell lines, MCF7-ADR (left) and MDA-MB-468 (right). Data are a mean ± S.D. (error bars) of three independent experiment in triplicate. ***p < 0.001; **p < 0.01; *p < 0.05.

To validate the miRNA microarray analysis, we explored the possibility that selected miRNAs were activated by transient overexpression of LY6K or ERα in each breast cancer cell. We first confirmed that all three primary-miRNA (pri-miRNA), activated by RNA Polymerase II binding to the relevant DNA sequence during miRNA biogenesis, were significantly increased (Figure 3A) and mature miRNA expression also increased (Figure 3B and 3C). These results revealed that selected miRNAs were transcriptionally activated by LY6K or ERα.

### miR-192-5p induced by LY6K suppresses ERα expression

**Figure 4:**
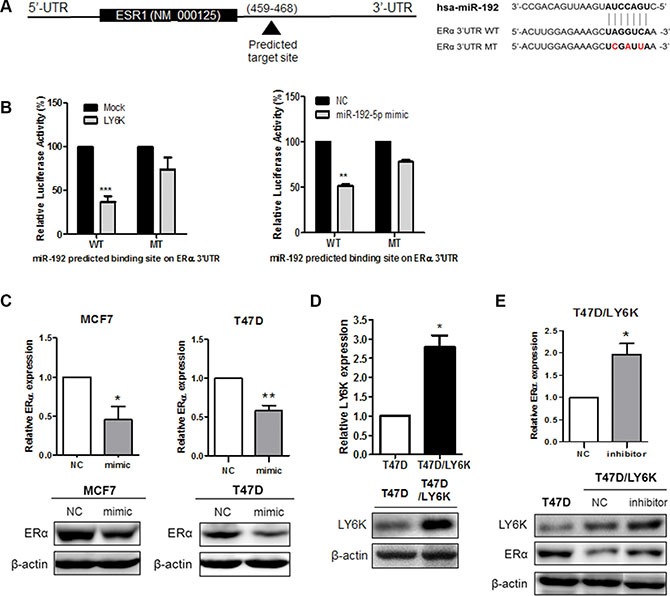
miR-192-5p directly binds ERα 3′UTR and regulates its expression (**A**) Gene structure of ESR1 showing the predicted target site of miR-192-5p in its 3′-UTR. (**B**) Luciferase assay in MCF7 cells showed miR-192-5p dependent repression of wild type (WT) ERα 3′UTR (left), whereas mutation (MT) of the miR-192-5p predicted binding site did not affect repression (right). (**C**) Re-expression of miR-192-5p repressed the mRNA and protein expression of ERα in MCF7 and T47D. (**D**) mRNA and Protein expression of LY6K was determined in Human LY6K over-expressing stable cell line by qRT-PCR and western blot. (**E**) Inhibition of miR-192-5p expression increases ERα expression in T47D/LY6K stable cells. Mimic, miR-192-5p mimic treated cell; inhibitor, miR-192 inhibitor treated cell. Data present mean ± S.D. (error bars) of three independent experiments in triplicate. ***p < 0.001; **p < 0.01.

To determine whether the reduced expression of ERα was due to direct targeting among miR-29b-3p, miR-29c-5p and miR-192-5p, we cloned luciferase reporter construct ESR1 3′-UTR containing the predicted binding site for each miRNA (Figure 4A and Supplementary Figure S2A). Among the selected miRNAs, only miR-192-5p altered luciferase activity in wild type constructs when compared with the mutant type construct. Likewise, luciferase activity was reduced by the transfection of LY6K (Figure 4B). In the case of miR-29b-3p and miR-29c-5p, luciferase activity was no different between the wild type reporter construct and in the mutant with regard to the predicted binding site following the transfection of each miRNA (Supplementary Figure S2B). In conclusion, the results of the dual luciferase assay confirmed that miR-192-5p directly targets ESR1.

To assess a possible role for the downregulation of ERα in MCF7 and T47D, we transfected either a miR-192-5p mimic (mimic) or a negative control mimic (NC). We confirmed the miR-192 expression using qRT-PCR after treating mimic (Supplementary Figure S3A). The mRNA and protein of ERα were significantly reduced by the overexpression of miR-192-5p in both MCF7 and T47D (Figure 4C). In addition, to observe whether the inhibition of miR-192-5p expression restored the mRNA expression of ERα, we generated a T47D stable cell line over-expressing human LY6K genes (T47D/LY6K) using G418 selection. It was confirmed that this stable cell line expressed plenty of LY6K mRNA and protein by qRT-PCR and western blot, respectively (Figure 4D). By using T47D/LY6K cells, we repressed the expression of miR-192-5p and confirmed the level of miR-192-5p by qRT-PCR (Supplementary Figure S3B). Consequently, inhibition of miR-192-5p restored ERα mRNA expression. Likewise, the protein expression of ERα was upregulated by inhibiting miR-192-5p expression in T47D/LY6K stable cells (Figure 4E). Taken together, miR-192-5p, induced by LY6K, repressed the level of ERα expression.

### miR-500a-3p, induced by ERα, targets and inhibits human LY6K expression

**Figure 5:**
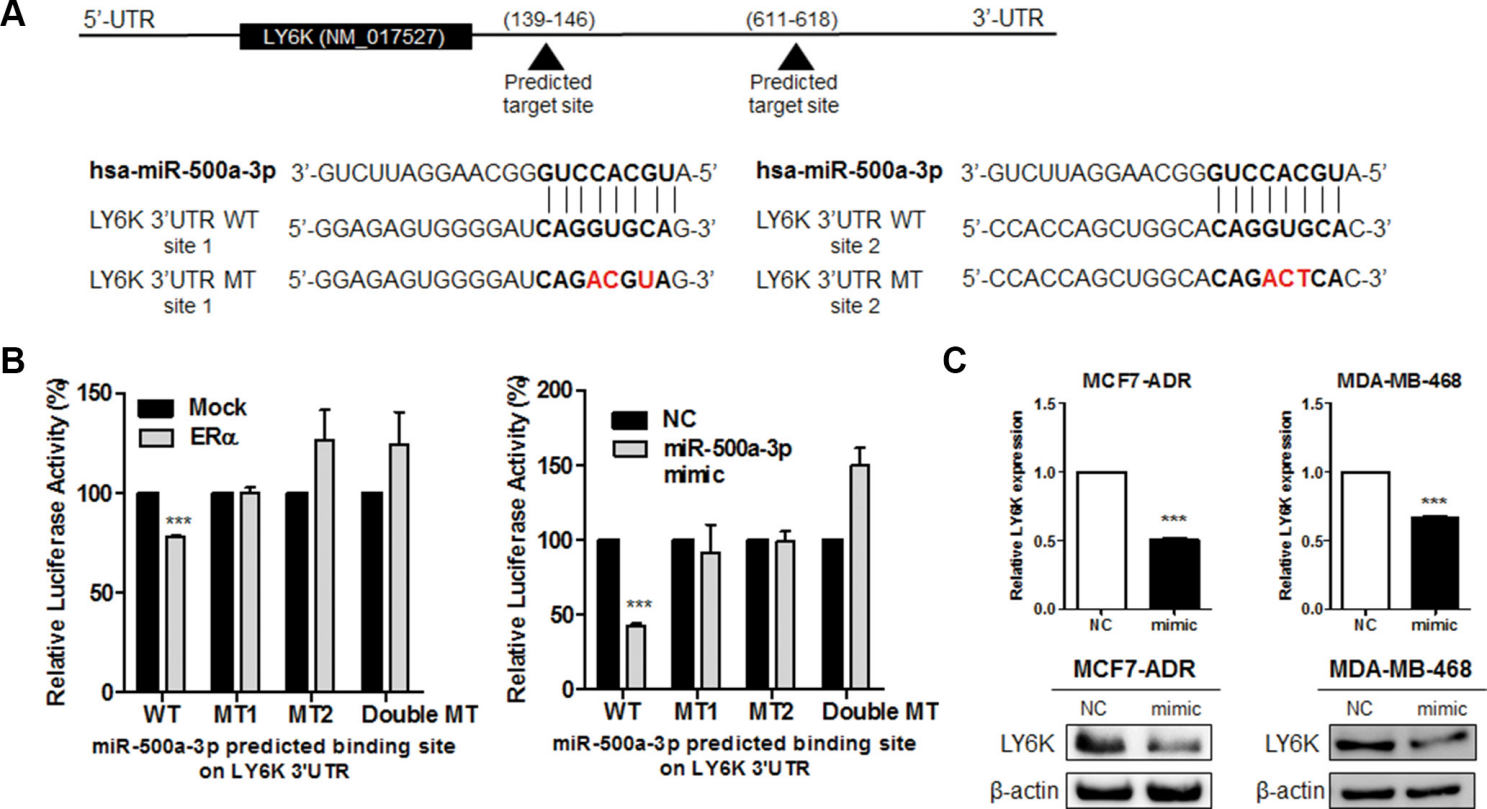
LY6K is a direct target of miR-500a-3p and down regulates its expression (**A**) Gene structure of LY6K showing the two predicted target sites of miR-500a-3p in its 3′UTR. (**B**) Dual Luciferase assay in HEK293T cells showed ERα (left) and miR-500a-3p (right) dependent inhibition of wild type (WT) LY6K 3′-UTR, whereas the mutant type (MT) seed sequence restored luciferase activity. (**C**) Ectopic expression of miR-500a-3p represses LY6K mRNA and protein expression in MCF7-ADR (left) and MDA-MB-468 (right). NC, miRNA negative control; mimic, miRNA mimic. Data represent mean ± S.D. (error bars) of three independent experiment in triplicate. ***p < 0.001.

In the same context, to determine whether the reduced expression of LY6K was due to direct targeting among three selected miRNAs, we searched the 3′UTR region of the LY6K gene for the miRNA binding motif using miRnada program. We generated luciferase reporter constructs with human LY6K 3′UTR containing each miRNA (Figure 5A and Supplementary Figure S4A). Among these miRNA, only miR-500a-3p overexpression led to a decrease in luciferase activity. Luciferase activity was also reduced by ectopic expression of ERα. This response was abrogated by the mutation of two miR-500a-3p targeted seed sequences (Figure 5B). The luciferase activity was no different between the wild type reporter construct and the mutant construct of the predicted binding site following the transfection miR-34a and miR-194-5p (Supplementary Figure S4B).

We further identified the possible role of LY6K downregulation by the overexpression of a miR-500a-3p mimic. We transfected the miR-500a-3p mimic (mimic) and the negative control mimic (NC). We confirmed that the transfection efficiency of the miR-500a-3p mimic using qRT-PCR (Supplementary Figure S3C). LY6K mRNA and protein expression was significantly reduced by ectopic miR-500a-3p in both MCF7-ADR and MDA-MB-468 (Figure 5C). These results suggested that miR-500a-3p, induced by ERα, directly repressed LY6K expression.

### Inhibition of miR-192-5p sensitizes resistance to tamoxifen in breast cancer cells

**Figure 6:**
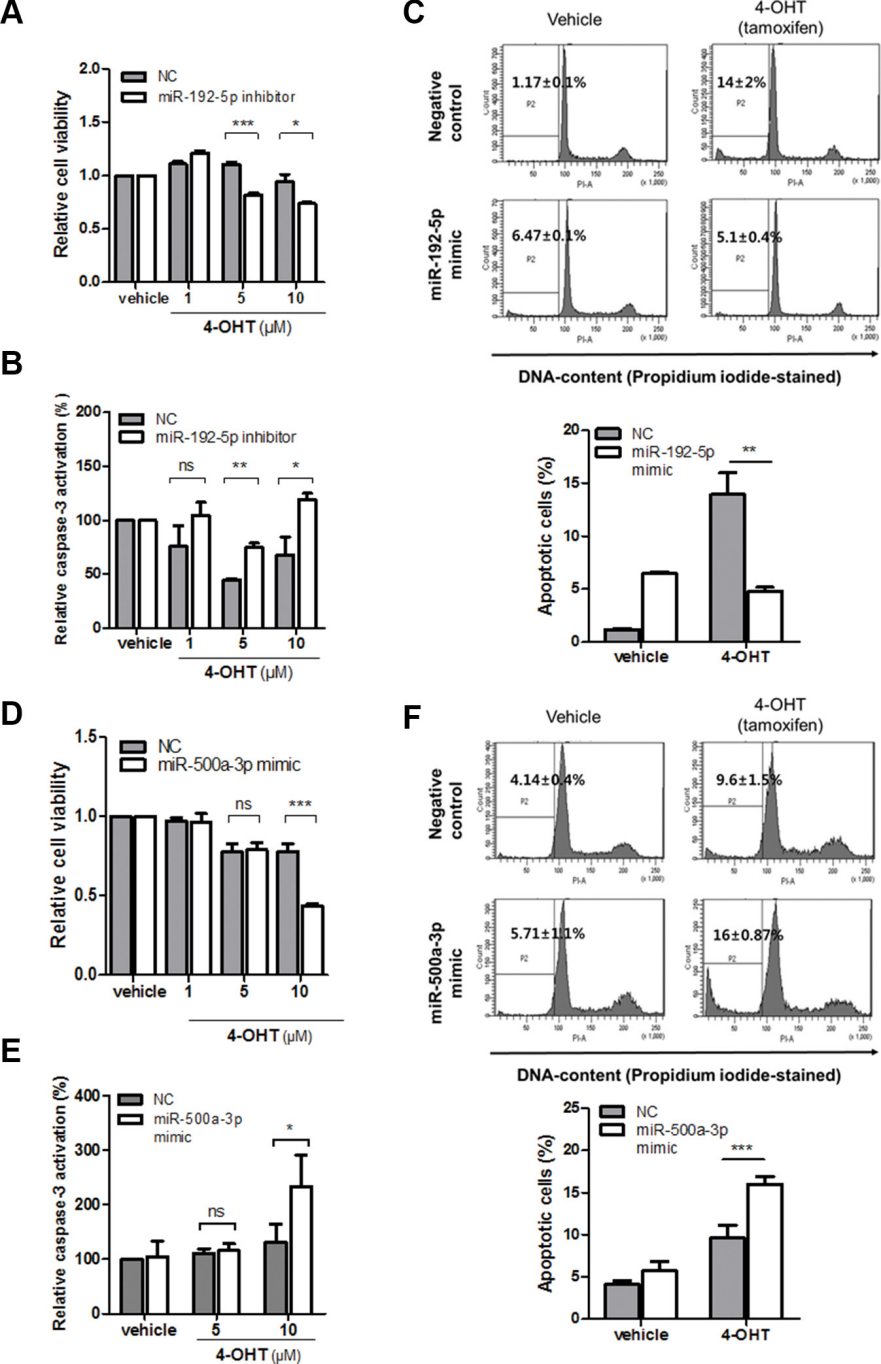
miR-192-5p and miR-500a-3p mediates tamoxifen sensitivity in breast cancer (**A**) Cell viability after transfection with miR-negative control inhibitor (NC) and miR-192-5p inhibitor in stably overexpressing LY6K (T47D/LY6K) cells was measured 3 hours after treatment with 4-OHT in a dose-dependent manner. (**B**) Caspase-3 activity was determined in T47D/LY6K transfected with miR-negative control inhibitor (NC) or miR-192-5p inhibitor after incubating with vehicle or 4-OHT (1, 5, 10 μM) for 3 hours. (**C**) T47D cells with miR-192-5p mimic transfected and then treated with 4-OHT (5 μM) for 24 hr. Apoptotic cells were assayed by flow cytometry sub-G1 analysis. (**D**) Cell viability with miR-negative control (NC) or miR-500a-3p was measured after 24 hr incubation with various doses of 4-OHT in MCF7-ADR. (**E**) Caspase-3 activation was increased by miR-500a-3p after 48 hr treatment with vehicle or 4-OHT. (**F**) MCF7-ADR cells with miR-500a-3p mimic transfected and then treated with 4-OHT (10 μM) for 24 hr. Apoptotic cells were determined by flow cytometry (sub-G1 analysis of PI staining). The graph shows the mean ± S.D. (error bars) of three independent experiments. ***p < 0.001; **p < 0.01; *p < 0.05; ns, non-specific.

Having demonstrated the miRNA-dependent reciprocal regulation of LY6K and ERα expression, we further studied whether miR-192-5p and miR-500a-3p are functionally involved in tamoxifen responsiveness in breast cancer. This is because tamoxifen is a selective estrogen antagonist and a well-known drug for breast cancer. To identify whether T47D/LY6K cells had tamoxifen resistance, we evaluated the cell viability after treating T47D and T47D/LY6K cell with tamoxifen. The cell viability was significantly reduced in T47D cells after treatment with tamoxifen whereas, the cell viability was barely reduced in T47D/LY6K because ERα expression was suppressed (Supplementary Figure S5A). Moreover, compared to the negative miRNA inhibitor (NC), inhibition of the miR-192-5p expression made T47D/LY6K cells more susceptible to tamoxifen (Figure 6A).

Tamoxifen was also reported to induce apoptosis and to inhibit cell proliferation in breast cancer cells [20]. After treating T47D and T47D/LY6K cells with tamoxifen, Caspase-3 activation was significantly increased in T47D cells but was relatively low in T47D/LY6K (Supplementary Figure S4B). Since the inhibition of miR-192-5p in T47D/LY6K affected cell viability, we further investigated the effect of tamoxifen-induced apoptosis by inhibiting the activity of miR-192-5p. Caspase-3 activity was increased by treatment with the miR-192-5p inhibitor in comparison with the negative miRNA inhibitor (NC) after incubating T47D/LY6K cells with tamoxifen (Figure 6B).

In addition, to confirm whether miR-192-5p enhancement induced tamoxifen resistance, we investigated that sub G1 phase of cell cycle after treatment of 4-OHT with miR-192-5p. The apoptotic cells was reduced by ectopic miR-192-5p in T47D cells (Figure 6C), suggesting that the miR-192-5p suppressed tamoxifen-induced apoptosis. From these results, miR-192-5p appears to regulate resistance to tamoxifen through cell viability along with cell apoptosis.

### miR-500a-3p enhances tamoxifen sensitivity by promoting apoptosis in breast cancer cells

We have shown that miR-500a-3p downregulates LY6K expression by directly targeting its 3′UTR. In this study, we investigated miR-500a-3p enforced tamoxifen sensitivity through the activation of apoptosis in ERα-negative cancer cells. This is because tamoxifen is effective in ERα-positive breast cancer patients but only 10~15% of ERα- negative patients respond to tamoxifen. We assessed cell viability by treatment with tamoxifen to transiently miR-500a-3p overexpression in MCF7-ADR. The cell viability was significantly reduced by ectopic expression of miR-500a-3p in MCF7-ADR (Figure 6D). In addition, we examined the effect of miR-500a-3p re-expression on enhancing tamoxifen sensitivity through apoptosis. miR-500a-3p significantly affected caspase-3 activation after treatment with tamoxifen (10 μM) compared to negative control (NC) (Figure 6E). Similar results were also found cells analysis in the sub-G1 phase of the cell cycle after treatment of tamoxifen using flow cytometry. Ectopic expression of miR-500a-3p was found to promote tamoxifen-induced apoptosis in MCF7-ADR (Figure 6F). The results together indicate that miR-500a-3p, induced by ERα, regulates tamoxifen sensitivity by targeting LY6K in ERα-negative breast cancer cells.

### miRNAs involved in the mechanism of LY6K and ERα are related to tamoxifen susceptibility in breast cancer patients

**Figure 7:**
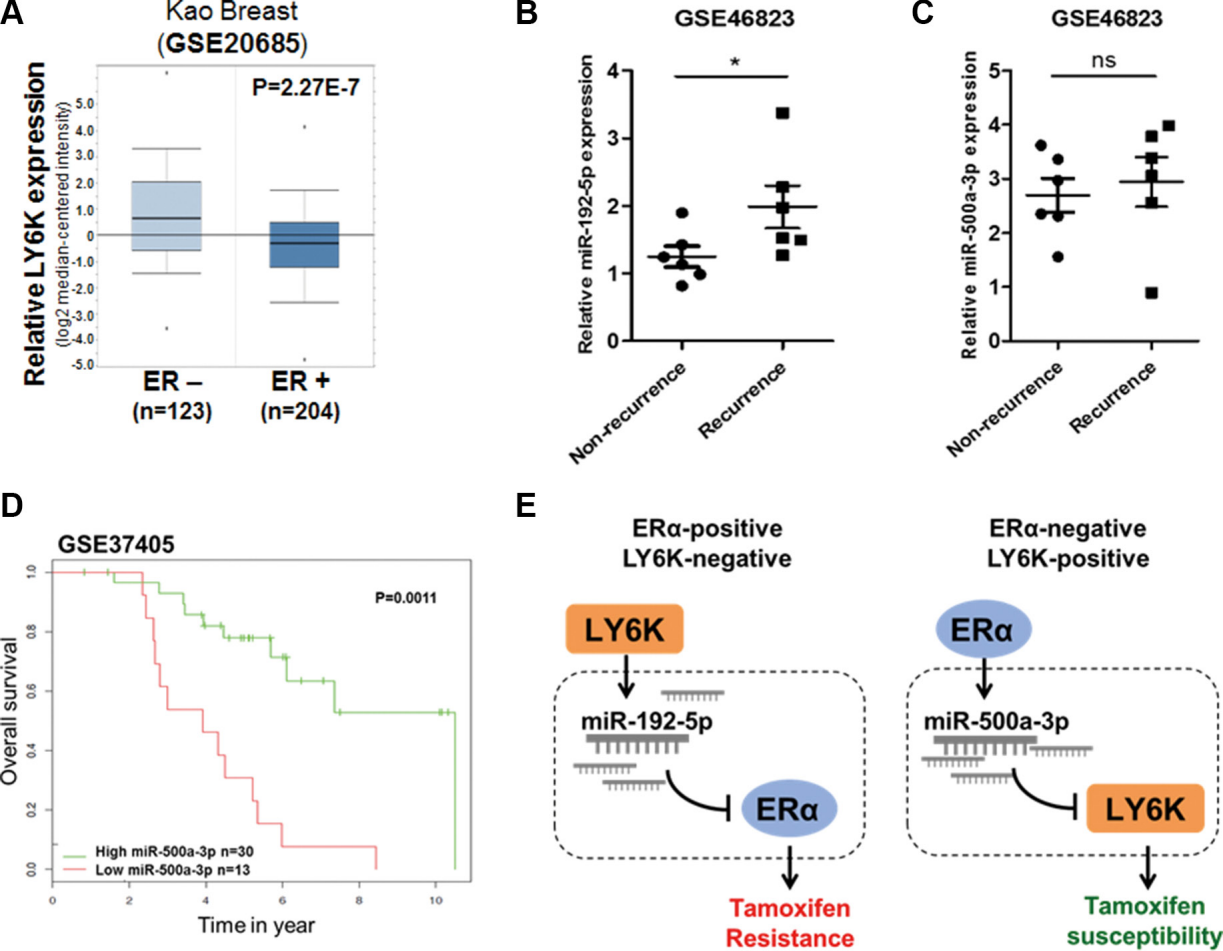
miR-192-5p and miR-500a-3p involved in LY6K and ERα are related to tamoxifen resistance in breast cancer patients (**A**) The relative LY6K expression depending on ER status in kao-breast cancer from oncomine™. (**B**) miR-192-5p expression in non-recurrence and recurrence primary breast tumors after tamoxifen treatment. The data were obtained from GSE 46823 (**C**) miR-500a-3p expression showed non-significant differences between non-recurrence and recurrence primary breast tumors after tamoxifen treatment. The data were obtained from GSE 46823. (**D**) Low expression of miR-500a-3p was correlated with poor survival outcomes in breast cancer patients received tamoxifen mono-therapy. (**E**) Hypothetical schematic pathway image. The mechanism for miR-192-5p and miR-500a-3p effects on tamoxifen susceptibility through the regulation of target genes in breast cancer. *P < 0.05; ns, non-significant.

We have shown that LY6K and ERα have an inverse correlation in breast cancer and that miRNAs involved in this mechanism affect tamoxifen resistance. To further ascertain whether miRNAs involved in the mechanism for LY6K and ERα are correlated with clinical outcomes in breast cancer patients, we first investigated the inverse correlation between LY6K and ERα expression in breast primary tumor samples obtained from oncomine™. ER positive tumors had relatively low LY6K expression while ER negative tumors showed high LY6K expression (Figure 7A). In addition, we observed miR-192-5p and miR-500a-3p expression in primary breast tumors treated with tamoxifen. Patients with recurrence showed increased miR-192-5p expression (Figure 7B). These results suggested that increasing expression of miR-192-5p could lead to recurrence in ERα-positive breast cancer patients because reduced expression of ERα is one of the causes of tamoxifen resistance. Although miR-500a-3p expression was not significantly different in recurrence (Figure 7C), expression of miR-500a-3p was significantly correlated with survival outcome in breast cancer patients treated with tamoxifen (Figure 7D).

In conclusion, the expression of miR-192-5p is up-regulated because of increased LY6K in ERα-positive and LY6K-negative breast cancer. The expression of miR-500a-3p is also up-regulated by ERα overexpression in LY6K-positive and ERα-negative breast cancer. Increased expression of miR-192-5p leads to decreased ERα expression, which is one of the problems that lead to tamoxifen resistance in ERα-positive cancer. In addition, upregulated miR-500a-3p expression, instead of ERα restoration, can affect tamoxifen susceptibility by targeting LY6K in ERα-negative breast cancer (Figure 7E).

## DISCUSSION

In our data, we clearly found that the expression of LY6K and ERα is negatively correlated in breast cancer cells. We observed the down-regulation of ERα expression when overexpressing the human LY6K gene in ERα-positive cells (Figure 1C and 1D). Among the different causes of resistance to hormonal therapies, the loss of ERα expression is an important factor in breast cancer [21]. This reduced expression of ERα brought by LY6K may provide a clue for patients that develop resistance to hormonal therapies. In addition, LY6K expression is downregulated by ectopic expression of ERα (Figure 1E and 1F). It was reported that highly upregulated LY6K is related to cell proliferation and metastatic abilities in breast cancer [10]. These findings suggested that ERα could be involved in attenuating metastatic and proliferative capacities and may enhance drug sensitivities in ERα-negative breast cancer.

It is widely known that miRNA is important as an oncogene or a tumor suppressor gene depending on the subtypes of a particular cancer [22]. Here, we showed that miR-192-5p, induced by LY6K, and miR-500a-3, induced by ERα, were selected through microRNA microarray (Figure 3 and Supplementary Figure S1). Elevated LY6K activated matrix metalloproteinase proteins MMP-2 and MMP-9 [23]. The expression of miR-192-5p is transcriptionally activated by TGF-beta, which well known as growth factor stimulated by MMPs [24, 25]. Although LY6K is not known as a transcriptional factor, transcription of miR-192-5p might be indirectly promoted by ectopic expression of LY6K. Recently, miR-192-5p was also identified as a potential target for esophageal cancer cells in a study profiling the development of chemotherapy resistance [26]. We observed that miR-192-5p was related to tamoxifen resistance through cell viability and apoptosis in breast cancer cells (Figure 6). In addition, miR-192-5p expression was increased in primary tumor samples treated with tamoxifen (Figure 7), so inhibition of miR-192-5p could be a potential therapeutic approach for the treatment of tamoxifen resistant breast cancer.

Furthermore, we determined that elevated ERα stimulates miR-500a-3p and suppress LY6K expression in ERα-negative breast cancer cells (Figure 3 and Figure 5). Many studies have attempted to restore ERα expression in order to increase tamoxifen sensitivity in ERα-negative breast cancer. HDAC inhibitors can induce re-expression of ERα transcriptional activity and improve endocrine therapy when combined with DNA-damaging agents [27]. CpG methylation of the ER promoter by inhibition of DMNT activity can reactivate ER because of transcriptional silencing in ERα-negative breast cancer cells [28, 29]. Similarly, our results suggested a novel mechanism involving the downregulation of LY6K via the miR-500a-3p axis, induced by ERα, and may be involved in attenuating metastatic and proliferate capacities in ERα-negative breast cancer. In this respect, upregulation miR-500a-3p may increase tamoxifen susceptibility because restoration of ERα leads to increase miR-500a-3p expression about 2~3 times (Figure 3C and Supplementary Figure 1). Although there are no significant differences in tamoxifen resistant tumor samples (Figure 7C), these results were not clearly in accord with the environment that miR-500a-3p activities are increased by overexpression of ERα. Also, low expression of miR-500a-3p is correlated to poor survival outcome in breast cancer patients who receiving tamoxifen mono therapy (Figure 7D). Thus, miR-500a-3p may help to increase tamoxifen susceptibility in breast cancer.

In conclusion, we showed for the first time that the involvement of miR-192-5p and miR-500a-3p regulates the mechanism for the interaction between ERα and LY6K and is related to tamoxifen responsiveness in breast cancer. These findings describe not only the loss of ERα correlated with miR-192-5p, induced by LY6K, in ERα-positive breast cancer but also how upregulation of miR-500a-3p affects tamoxifen-induced cell death in ERα-negative breast cancer. These results provide clues that not only the molecular mechanism of LY6K and ERα in breast cancer but also the inhibition of miR-192-5p in ERα-positive breast cancer and the overexpression of miR-500a-3p in ERα-negative breast cancer could be effective therapeutic agents.

## MATERIALS AND METHODS

### Cell culture and plasmid transfection

MCF7 and MDA-MB-468 cells were provided by Sapporo Medical University in December, 2010. Human breast carcinoma MCF7-ADR cell was obtained from Dr. YM Park (Roswell Park Cancer Institute, Buffalo) in 2006. These cells were tested by short tandem repeat marker for DNA fingerprinting analysis (Korean Cell Line Bank); Both MCF7 and MCF7-ADR were lastly tested in August, 2014; MDA-MB-468 in September, 2014. T47D cells were purchased from ATCC (Manassas, VA) in January, 2015. T47D cell was characterized and authenticated in the Cell Biology collection using short tandem repeat DNA profiles. Breast cancer cells experimented in this study were maintained in Dulbecco's modified Eagle's medium (WELGENE Inc, Korea) supplemented with 10% fetal bovine serum (FBS) (Gibco®, USA) in a 37°C humidified incubator and an atmosphere of 5% CO2. A T47D stable cell line overexpressing the human LY6K gene was produced by G418 selection. Firstly, selected concentration for stable cells was 2mg/ml. After selection, G418 concentration was reduced from 1 mg/ml to 500 ug/ml and then 200 ug/ml. The maintaining concentration of G418 was 200 ug/ml.

MCF7 and T47D were transfected for 48 hrs with hLY6K/pCMV6-Entry clone purchased from Origene. MCF7-ADR and MDA-MB-468 were transfected with human ERα/pCMV plasmid using Fugene^®^ reagent (Promega) according to the manufacturer's instructions. After 48 hours, the breast cancer cells were harvested and separated for qRT-PCR and Western blot analysis.

### Co-transfection with plasmid and small interfering RNA

MCF7 and T47D cells were transfected with Argonatue 2 siRNA (SMARTpooled, Dharmacon) and human LY6K clone purchased from Origene Technologies using Lipofectamin 2000 (Invitrogen, USA) for 48 hours following by the manufacturer's instructions. MCF7-ADR and MDA-MB-468 cells were co-transfected with Argonatue 2 siRNA (Dharmacon, USA) and human ERα clone in the same manner. Transfection of both scrambled siRNA (Dharmacon, USA) and pCMV-Flag plasmid was used as the control experiments.

### Transfection of miRNA mimics and inhibitor

MCF7 and T47D were reverse-transfected with a miRVana™ miR-192p mimic (Ambion, USA) at a final concentration of 15 nM or 30 nM using the siPORT™ NeoFX™ Transfection agent (Ambion, USA) according to the manufacturer's instructions. MCF7-ADR and MDA-MB-468 cells were reverse-transfected in the same manner. The control experiments were transfected with a miRVana™ miR-negative control mimic (Negative control #1, Ambion, USA).

### Total RNA isolation and miRNA RT-PCR

Total RNAs for the miRNA-microarray were isolated using Trizol^®^ (Ambion, USA) as described in the manufacturer's instructions. We extracted total RNA including miRNA and other small RNAs from cultured cells with the miRNeasy RNA isolation kit (Qiagen). 500 ng of total RNA was reverse transcribed with the TaqMan^®^ MicroRNA Reverse Transcription Kit (Applied Biosystems) for has-miR-192-5p or has-miR-500a-3p detection.

### Quantitative RT-PCR (qRT-PCR)

Quantitative RT-PCR for miRNA expression analysis was performed with the TaqMan^®^ Universal PCR Master Mix (Applied Biosystems) using the LightCycler^®^ System (Roche) and RNU48 was used as an internal control. Quantitative RT-PCR for mRNAs were extracted using the NucleoSpin^®^ RNA/Protein kit (MACHEREY-NAGEL). 5 μg of mRNAs were reverse-transcribed by using M-MLV Reverse Transcriptase (Promega, USA), 10 pM oligo-dT, 2.5 nM dNTP mixture and RNAse inhibitor and each target gene was detected by using specific primers. PCR primer sequence were Human ERα, forward 5′- GGCCCAGCTC CTCCTCATG - 3′and reverse 5′ – AGTGGCTTTGGTCCGTCTCC - 3′; Human LY6K, forward 5′- AGCCCATGCCCTTCTTTTAC -3′ and reverse 5′ – CCAGCCACAGCCCACCACAG - 3′; Human Argonaute 2 (Ago2), forward: 5′ – CTAGACCCGACTT TGGGACCT - 3′ and reverse 5′– GGGCACTTCTCTGGC TTGATA -3′; Human 18s rRNA, forward 5′-GTCGGCG TCCCCCAACTTCTT -3′ and reverse 5′- CGTGCAGC CCCGGACATCTA -3′ Human 18s rRNA was used as a housekeeping gene.

### Western blotting

Proteins were isolated using the NucleoSpin^®^ RNA/Protein kit (MACHEREY-NAGEL). Protein contents were measured using Bicinchoninic acid Solution (Sigma, USA) and Copper (II) sulfate solution (Sigma, USA). Protein were separated on 12% or 8% SDS-PAGE gel. Western blotting of SDS-PAGE gels was performed using standard methodology. Primary antibodies were diluted at 1:1000 in 1% skim milk in PBST. Primary antibodies used in this study were LY6K (Santa Cruz Biotechnology, USA), ERα (Santa Cruz Biotechnology, USA), Argonate2 (abcam^®^, UK) and β-actin (Bethyl Laboratories, US). β-actin was used as a loading control. Immunoreactive proteins were detected by horseradish peroxidase-conjugated secondary antibodies and the enhanced chemiluminescence reagent, EzWestLumi plus (ATTO, JAPAN).

### Dual-Luciferase reporter assay

The 3′UTR of human ERα was cloned into the XbaI site of the pGL3-control vector (Proemega, USA). The seed sequence of miR-29b-3p, miR-29c-5p and miR-192-5p on human ERα 3′UTR was mutated by with a PCR-based approach. We transfected luciferase constructs (1.8 μg of reporter gene/well in 6-well plate) and either human LY6K clones (200 ng/well in 6-well plate) or 30 nM of miRNA mimics into MCF7 cells using Lipofectamin 2000 (Invitrogen, USA). Human LY6K 3′UTR that included the predicted miR-500a-3p seed sequences into psiCHECK™-2 vector (Promega, USA) using the In-fusion^®^ HD Cloning Kit (Clontech Laboratories, USA). The seed sequence for miR-500a-3p on LY6K 3′UTR was mutated by using the QuickChange II XL Site-Directed Mutagenesis Kit (Agilent Technologies, USA). We transfected HEK293T cells with the luciferase reporter constructs containing the LY6K 3′UTR variants and 30 nM of miR-500a-3p mimics or negative control miRNA using Lipofectamin 2000 (Invitrogen, USA). After 48 hours, all experimental cells were passively lysed and measured with the Dual Luciferase Assay System (Promega, USA).

### Cell viability assay

T47D and T47D/LY6K cells were seed at 1 × 10^4^ cell/well in 96-well plates. Cell were incubated with 4-Hydroxytamoxifen (4-OHT; Sigma, USA) for 3 hr. T47D/LY6K cells were seeded and transfected with miR-192-5p inhibitor or a negative control inhibitor at a final concentration of 30 nM using siPORT™ neoTX™ transfection Agent (Ambion, USA). MCF7-ADR and MDA-MB-468 cells were seeded and transfected with miR-500a-3p mimic or negative control mimic at a final concentration of 15 nM. After 24 hours, the cells were re-seeded in 24-well plates at a density of 2 × 10^5^ per well then treated with a range of 4-OHT concentrations from 1 to 10 μM, and incubated for 48 hours. The WST-8 (Enzo^®^, USA) labeling mixture was added to each well and quantified using a scanning 96-well spectrophotometer according to the manufacturer's protocol.

### Caspase-3 activation assay

T47D and T47D/LY6K were treated with vehicle or 4-OHT and then incubated for 3 hr. T47D/LY6K cells were transfected with miR-192-5p inhibitor or negative control then treated with 4-OHT. MCF7-ADR cells were seeded in 100 mm dishes with miR-500a-3p mimic or negative control mimic. Then, 4-Hydroxytamoxifen (4-OHT) was treated at final concentration of 30 nM. After 48 hours, cells were lysed and the cell lysates were employed to measure the activity of a Caspase-3 using Caspase-3/CPP32 Colorimetric Assay Kit (Biovision, USA) according to the manufacturer's instructions.

### Cell apoptosis by flow cytometry analysis

Drug-induced apoptotic cell death was analyzed using measurement of apoptotic cell by flow cytometry. Briefly, cells were treated with tamoxifen and incubated for 24 hr. Cell were collected and washed ice-cold 1 X PBS and then fixed with cold 70% ethanol overnight. Fixed cell stained with 20 μg/ml propidium iodide in PBS containing 100 μg/ml RNase A for 30 minutes and then analyzed by FACSCanto II flow cytometry (BD biosciences, USA).

### Analysis of patient data

The mRNA data set for the comparison of LY6K and ERα expression obtained from the Oncomine™ (Compendia Bioscience, Ann Arbor, MI) was used for analysis and visualization. The miRNA expression analysis in ER+ tumors after tamoxifen treatment was obtained from the NCBI GEO database (GSE 46823 [30]). The data on overall survival of breast cancer patients receiving adjuvant Tamoxifen mono-therapy was acquired from drugsurv (http://www.bioprofiling.de/GEO/DRUGSURV/index.html) using the NCBI GEO database (GSE37405 [31]).

### Statistical analysis

All experiments were repeated at least three times in this study. One-tailed *t*-test was performed by GraphPad InStat (Graphpad software, La Jolla, CA, USA). Values were reported as means ± SD. *P* < 0.05 was considered significant. (*P < 0.05; **P < 0.01; ***P < 0.001; ****P < 0.0001).

## SUPPLEMENTARY FIGURES


