# Supplementary material for: miRNAs involved in LY6K and estrogen receptor α contribute to tamoxifen-susceptibility in breast cancer
Source: Oncotarget. 2016 Jun 11;7(27):42261–73. doi: 10.18632/oncotarget.9950 (PMC5173133; doi:10.18632/oncotarget.9950)
Supplement: Supplementary file 1 [file oncotarget-07-42261-s001.pdf]

miRNAs involved in LY6K and estrogen receptor α contribute to tamoxifen-susceptibility in breast cancer

Supplementary Materials

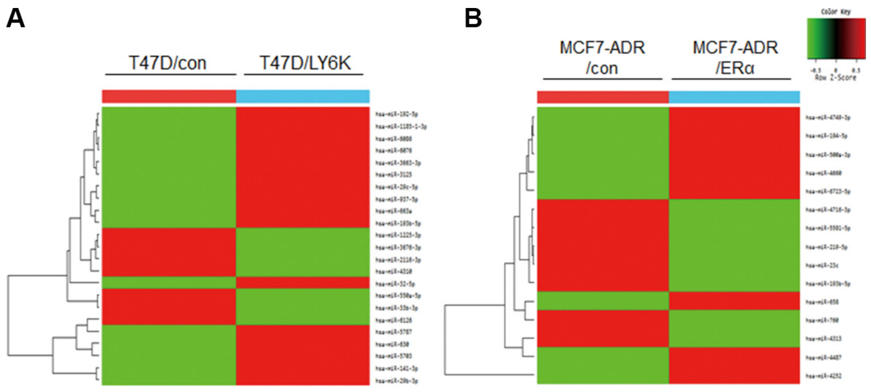

A

Probe ID	Fold change
hsa-miR-3125	1.55
**hsa-miR-29c-5p**	1.59
hsa-miR-3663-3p	1.61
hsa-miR-193b-5p	1.66
hsa-miR-937-5p	1.67
**hsa-miR-29b-3p**	1.72
hsa-miR-141-3p	1.80
hsa-miR-1185-1-3p	1.91
hsa-miR-5787	1.99
hsa-miR-663a	1.99
**hsa-miR-192-5p**	2.07
hsa-miR-5703	2.21
hsa-miR-6086	2.29
hsa-miR-6076	2.32
hsa-miR-630	2.37
**hsa-miR-32-5p**	8.10

B

Probe ID	Fold change
hsa-miR-4252	1.56
**hsa-miR-194-5p**	1.6
hsa-miR-658	1.61
hsa-miR-4749-3p	1.79
hsa-miR-4487	1.82
hsa-miR-6723-5p	1.86
**hsa-miR-500a-3p**	1.99
hsa-miR-4660	3.06
**hsa-miR-34a**	1.31

Supplementary Figure S1: The heat map of miRNA microarray. (**A**) The hierarchical clustering data was based on miRNA microarray analysis of T47D/Mock compared with T47D/LY6K cells. Upregulated miRNAs by LY6K were listed below heat map. (**B**) The hierarchical clustering data was based on miRNA microarray analysis of MCF7- ADR/Mock and MCF7-ADR/ERa. Elevated miRNAs by ERa were presented below heat map. Bold character means selected miRNA for further study.

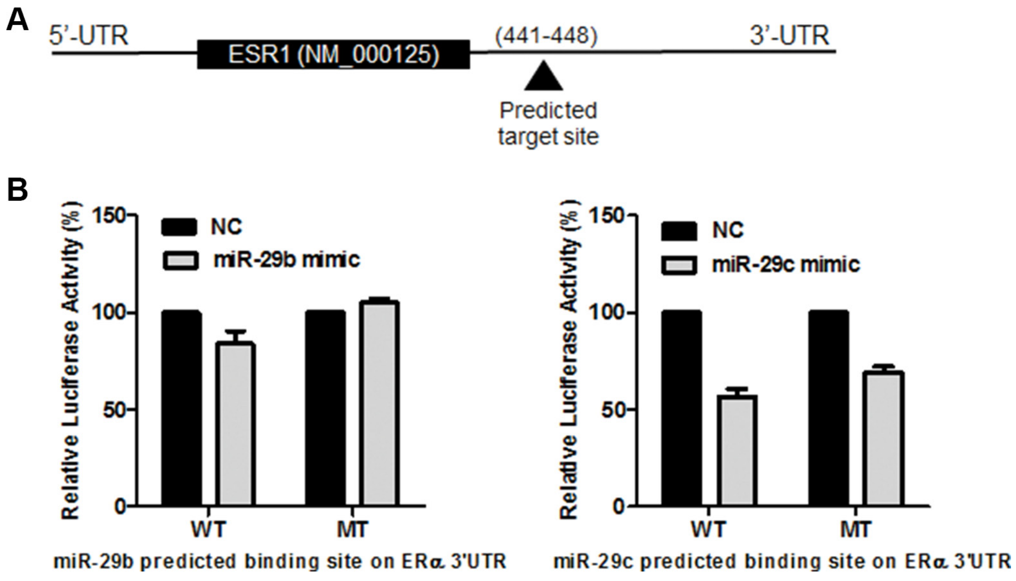

Supplementary Figure S2: miR-29b-3p and miR-29c-5p did not target ESR1 3′UTR. (**A**) Prediction of miR-29b-3p and miR-29c-5p binding site on ESR1 3′-UTR from miRanda (http://www.microrna.ora/). (**B**) Dual-luciferase assay in MCF7 cells show that miR-29b-3p and miR-29c-5p had little difference in WT and MT at each predicted binding site. Data present mean ± SD of three independent experiments in triplicate.

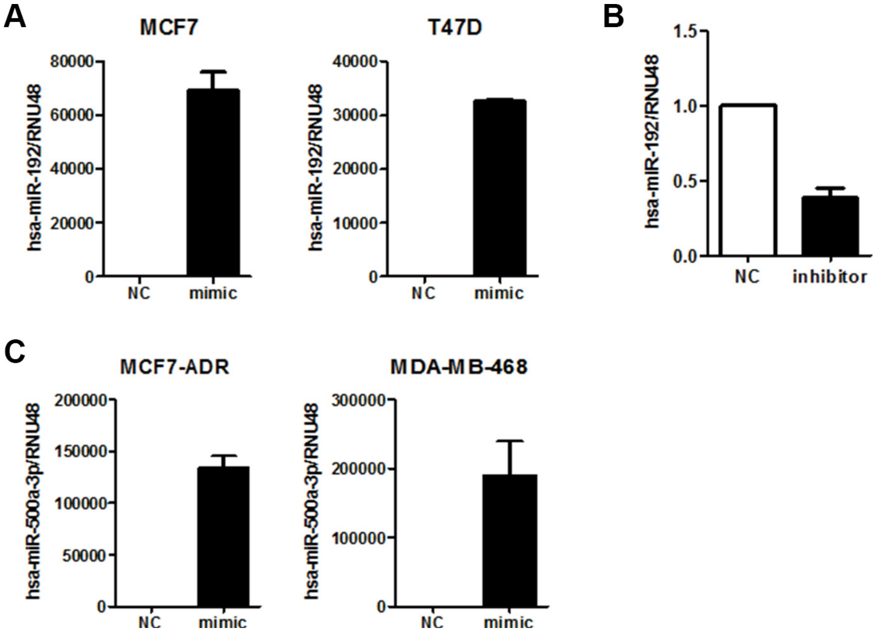

Supplementary Figure S3: The expression of miR-192-5p and miR-500a-3p after transfection in breast cancer cells. (**A**) The expression of miR-192-5p mimics in MCF7 and T47D. (**B**) The expression of miR-192-5p inhibitor in T47D/LY6K cells stably expressed with LY6K. (**C**) The expression of miR-500-3p mimics in MCF7-ADR and MDA-MB-468.

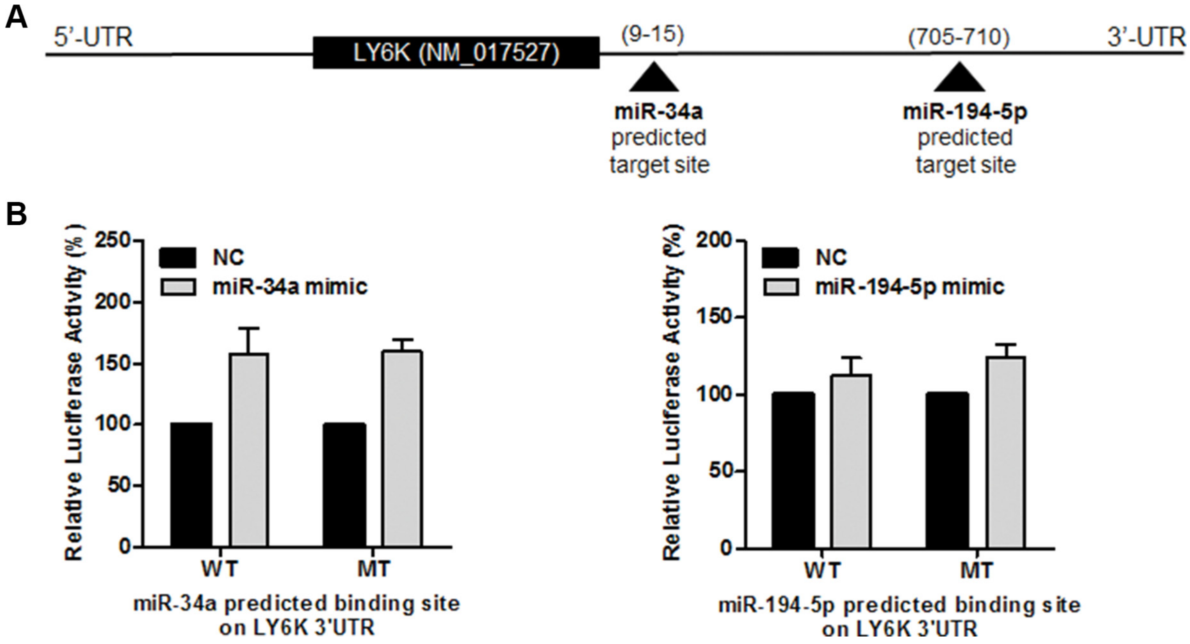

Supplementary Figure S4: miR-34a and miR-194-5p did not target LY6K 3′UTR. (**A**) Gene structure of LY6K 3′UTR with miR-34a and miR- 194-5p predicted binding site. Information of wild type structure (WT) and mutant type in miRNA binding site (MT). (**B**) miR-34a (left) and miR- 194-5p (right) do not affect luciferase activity in HEK293T cells as confirmed through a Dual luciferase assay.

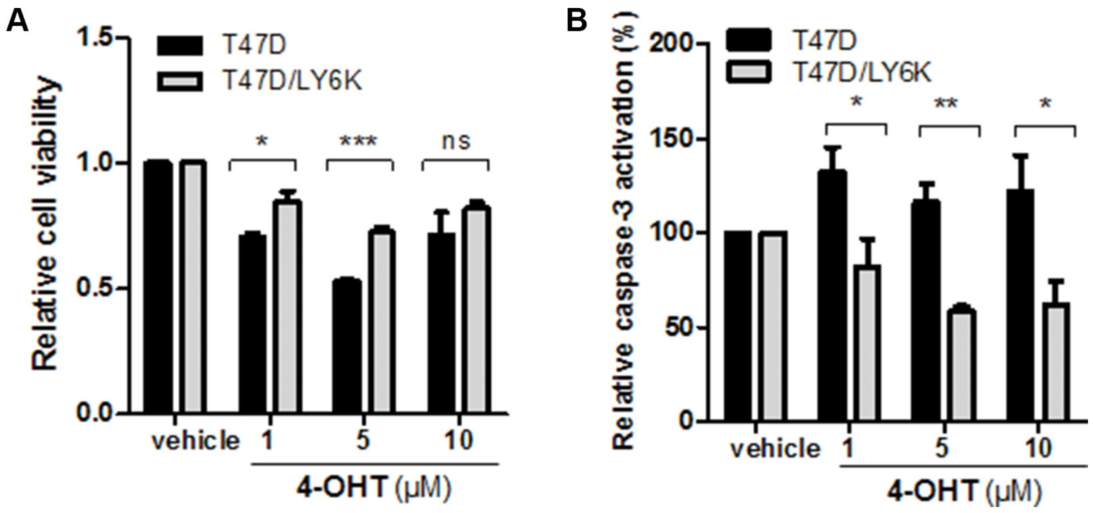

Supplementary Figure S5: T47D/LY6K cells show tamoxifen- resistance through Cell viability and apoptosis. (**A**) Cell viability of T47D and T47D with stably overexpressing LY6K (T47D/LY6K) cells was measured 3 hours after treatment with 4-OHT in a dose-dependent manner. (**B**) Caspase-3 activity is decreased only in T47D/LY6K cells after incubating with vehicle or 4-OHT (1, 5, 10 μM) for 3 hours.
